# Association of retinal microvascular abnormalities with cardiovascular diseases in patients with severe carotid artery stenosis

**DOI:** 10.7150/ijms.126670

**Published:** 2026-03-17

**Authors:** Li Zhang, Qi-Bo Ran, Chun-Yan Lei, Fei-Peng Jiang, Tian-Yu Yang, Zhi-Hao Xiao, Sheng Gao, Mei-Xia Zhang

**Affiliations:** Department of Ophthalmology, Sichuan University West China Hospital, Chengdu; Address: No. 37 Guoxue Lane, Chengdu, Sichuan Province, China, 610041.

**Keywords:** retinal microvascular abnormalities, carotid artery stenosis, cardiovascular diseases

## Abstract

**Purpose:**

To investigate and summarize the characteristics of retinal microvascular abnormalities (RMAs) in patients with severe carotid artery stenosis (CAS) and explore the association between the presence of RMAs and cardiovascular diseases (CVD).

**Methods:**

Color fundus photography and wide-field swept-source optical coherence tomography angiography (SS-OCTA) examinations were performed on all participants. Images were reviewed to identify RMAs. The occurrence and characteristics of RMAs were summarized. CAS patients with or without CVD were divided into different groups, and the association between the presence of RMAs and CVD were explored using multivariable logistic regression analysis.

**Results:**

This observational, cross-sectional study included 258 eyes from 129 patients with severe CAS. A total of 39/129 patients (30.2%) were identified with RMAs. These RMAs included branch retinal artery occlusion, asymptomatic retinal emboli, cotton wool spots, retinal hemorrhage, branch retinal vein occlusion, inner retinal layer hyperreflectivity, retinal nonperfusion and neovascularization of the optic disk. Multivariable logistic regression analysis revealed that RMAs was significantly associated with the presence of CVD in patients with severe CAS.

**Conclusions:**

This cross-sectional study summarized the characteristics of RMAs associated with CAS and identified RMAs as biomarkers of CVD in patients with severe CAS. Patients with RMAs were at high risk and should be carefully managed.

## Introduction

Cardiovascular diseases (CVD) are a group of disorders of the heart and blood vessels which is the leading cause of death globally. It's been estimated that 17.9 million people died from CVD in 2019, and 23.6 million annual CVD deaths were predicted to occur by 2030 1, 2. It's been found that patients with carotid artery stenosis (CAS) have high cardiovascular events rates and could benefit from specific interventions to prevent CVD events 3, 4. To identify high-risk patients among patients with CAS is an important issue that needs to be addressed. The eye is the unique “window” for observing vascular change within the body. Studies have proved the ability of ocular vascular findings in diagnosing and predicting non-ophthalmic diseases 5-7. With the advances in ocular imaging devices, the retinal microvasculature could now be accurately assessed and may serve as novel biomarkers for identifying patients at risk.

This study aims at summarizing ocular fundus findings in patients with severe CAS and exploring the association between retinal microvascular abnormalities (RMAs) and the presence of CVD in patients with severe CAS.

## Methods

### Study design and participants

This prospective, single-center, observational cohort study was implemented by the Department of Ophthalmology at West China Hospital, Sichuan University, Sichuan, China, from September 1st, 2023 to December 20th, 2024. The study was designed and performed following the ethical tenets of the 1964 Helsinki Declaration and proved by the Institute Ethics Committee of West China Hospital with verifiable consent (Approval number:20231171). All patients provided written informed consent before participation in this study. The details of the study design and patient recruitment have been reported elsewhere 8. Briefly, this cross-sectional study consecutively included patients with severe CAS (> 70%). Patients complicated with ocular diseases or neurological disorders were excluded. Patients with axial length > 26mm or diopter < -6D, or poor image quality were also excluded. All patients underwent detailed ophthalmic examinations including best-corrected visual acuity (BCVA), slit lamp bio-microscopy, intraocular pressure (IOP), axial length, color fundus photography (CFP), optical coherence tomography (OCT), and optical coherence tomography angiography (OCTA) with a 16*16 mm volume. Clinical characteristics and medical records were also recorded.

Subjects were classified into the CVD group if they had a diagnosis of coronary heart disease, congestive heart failure, myocardial infarction, atrial fibrillation or stroke, and others were classified into the control group.

### Imaging protocols

All participants underwent detailed ocular examination, including BCVA, IOP (TX-20, Canon, Tokyo), axial length (IOL Master Advanced Technology, Carl Zeiss, Meditec, Dublin, CA), slit lamp examination, CFP (CLARUS 500^TM^ or Daytona, Optos), OCT and OCTA (BM-400K BMizar, TowardPi Medical Technology, Beijing, China). OCTA scans were obtained using a 400 kHz SS-OCTA instrument (BM-400K BMizar, TowardPi) with a 16*16 mm volume. Each type of examination was completed by the same appointed operator.

### Identification of RMAs

CFP and OCTA images of both eyes were reviewed by two experienced retina specialists and RMAs were identified with the presence of any retinal abnormalities.

### Statistical analysis

All analyses were conducted using SPSS version 26 (SPSS, Inc., Chicago, IL, USA) and Microsoft Excel (version 16, Microsoft Corp, Redmond, WA, USA). Baseline characteristics of subjects are described using mean and SD for continuous variables and frequency with percentage for categorical variables. Demographics were compared for cases and controls using the Pearson χ2 test for categorical variables and the Student t test for continuous variables. We used multivariable logistic regression models to evaluate the relationship between the presence of RMAs and CVD. Covariates included age, sex, BMI, hypertension, diabetes, hyperlipidemia, smoking and drinking. P values < 0.05 were considered statistically significant.

## Results

### Demographic characteristics

A total of 258 eyes from 129 patients (115 males and 14 females) with severe CAS were included in the preoperative imaging analysis with a mean age of 66.38 ± 9.11 years and mean BMI of 24.0 ± 2.73. Among them, 65 patients (50.4%) presented with CVD, 53 (41.1%) with diabetes mellitus, 85 patients (65.9%) with hypertension, 24 patients (18.6%) with hyperlipidemia. 75 patients (58.1%) have a history of smoking and 62 patients (48.1%) of drinking. Mean systolic blood pressure was 134.9 ± 18.3 mmHg, and mean diastolic blood pressure was 81.07 ± 10.3 mmHg. Mean best-corrected logarithm of the minimum angle of resolution (LogMAR) visual acuity was 0.08 ± 0.16 and 0.06 ± 0.11 for the ipsilateral and contralateral eye, respectively, with no significant difference (p=0.113). Mean axial length was 23.8 ± 0.84 and 23.8 ± 1.11 for the ipsilateral and contralateral eye, respectively, with no significant difference (p=0.193). Mean intraocular pressure (IOP) of the ipsilateral eye was significantly lower than the contralateral eye (13.5 ± 2.8 mmHg vs14.3 ± 3.3 mmHg, p<0.001). 53 patients (41.1%) presented with visual symptoms, including 35 (27.1%) with amaurosis fugax, 15 (11.6%) with decreased visual acuity and 6 (4.7%) with visual field defect (Table 1).

**Figure 1:**
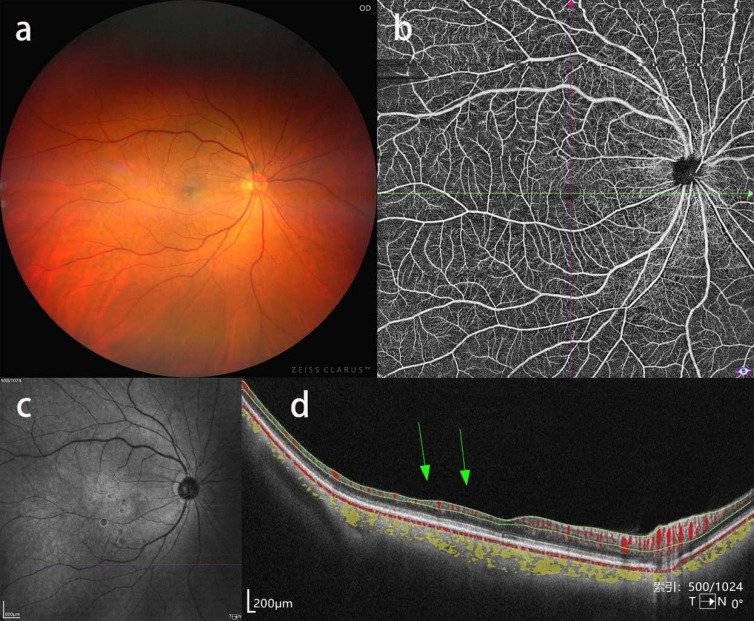
Ipsilateral BRAO in a patient with severe CAS. a, b, c: CFP, OCTA blood flow image and near-infrared fundus image showing no obvious abnormalities. d: corresponding b-scan image showing atrophy of inner retinal layers in the temporal macular region.

**Figure 2:**
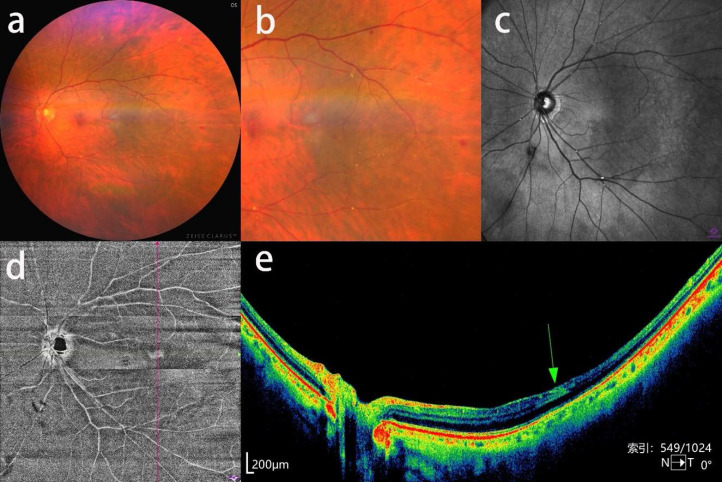
Asymptomatic retinal emboli, retinal hemorrhage and inner retinal hyperreflectivity in a patient with severe CAS. a, b: CFP and an enlarged view of multiple Hollenhorst plaques and focal retinal hemorrhage. c: near-infrared fundus image showing distinct white Hollenhorst plaques resting at the bifurcation of retinal arteries. d: OCTA enface image showing a wedge-shaped hyperreflective lesion at the temporal macular region. e: corresponding b-scan image showing hyperreflectivity of the inner retinal layer (green arrow) and reduced reflective signal of the retinal structure beneath the lesion.

**Figure 3:**
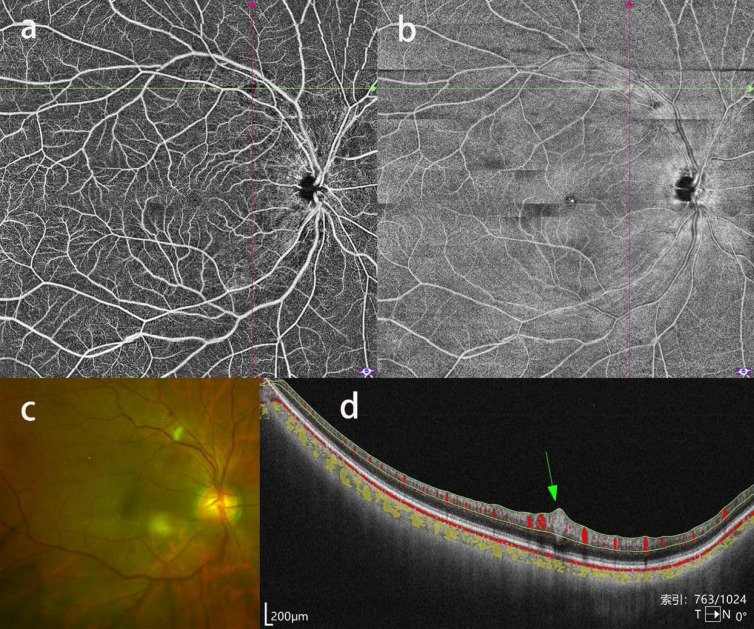
Cotton wool spots in a patient with severe CAS. a, b: OCTA blood flow image showing a focal blood flow signal defect and enface image showing a corresponding hyperreflective lesion at the superior temporal vascular arcades. c: CFP showing a corresponding yellow-white wedge-shaped lesion. d: b-scan image showing a fusiform lesion conforming to cotton wool spots.

**Figure 4:**
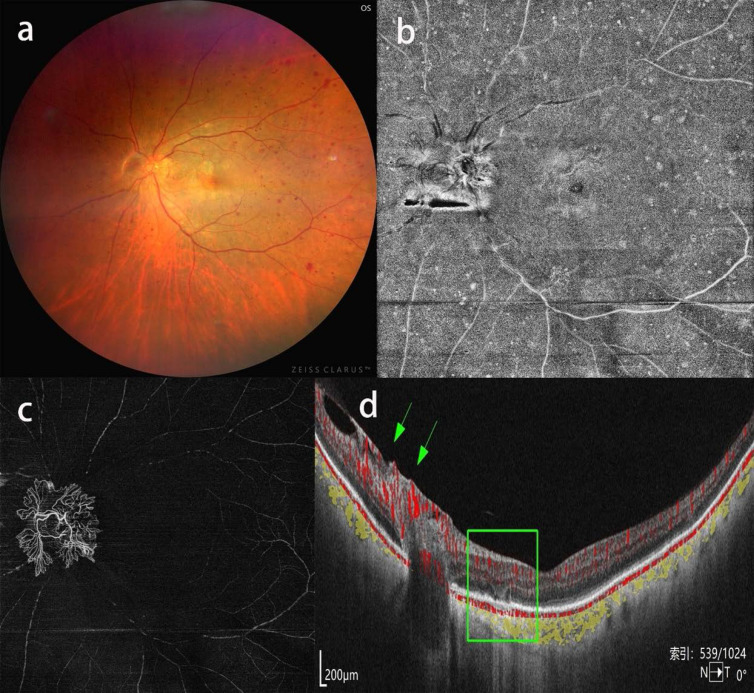
Neovascularization of the optic disk in a patient with severe CAS. a, b: CFP and OCTA enface image showing optic disk neovascularization and scattered retinal hemorrhage spreading the mid-peripheral fundus, c: OCTA blood flow image of the vitreous body showing thick neovascularization of the optic disk extruding into the vitreous body. d: b-scan image showing neovascularization of the optic disk with blood flow signal (green arrow) and collapse of focal outer retinal layers (green rectangle).

**Figure 5:**
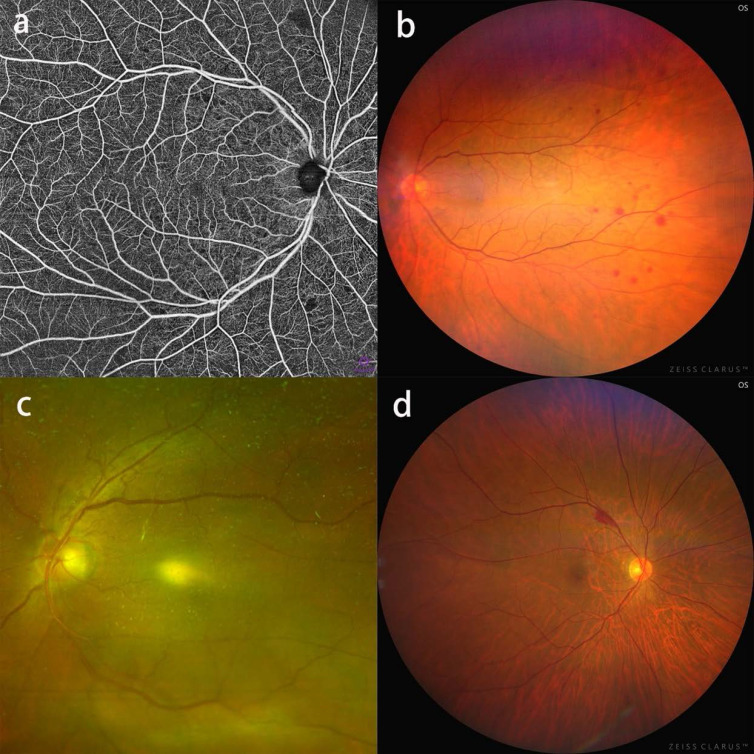
RMAs in patients with severe CAS. a: OCTA blood flow image showing flakey nonperfusion areas; b: CFP showing mid-peripheral deep retinal hemorrhage; c: CFP showing branch retinal vein occlusion at the superior optic disk region. d: CFP showing superficial retinal hemorrhage at the superior temporal vascular arcades

After reviewing all the CFP and OCTA images, RMAs associated with severe CAS were identified with consensus. In total, 39 patients (30.2%)/43 eyes were found with RMAs. Among them, 6/129 (4.7%) patients were identified with branch retinal artery occlusion (BRAO), 5 in the ipsilateral eyes and 1 in the contralateral eyes (Figure 1). 9/129 (7.0%) patients were identified with asymptomatic retinal emboli (ARE), 8 in the ipsilateral eyes and 1 in the contralateral eyes (Figure 2). 6/129 (4.7%) patients were identified with cotton wool spots, 5 in the ipsilateral eyes and 1 in the contralateral eyes (Figure 3). Retinal hemorrhage was found in a total of 21/129 (16.3%) patients, including 3 with superficial retinal hemorrhage and 18 with deep retinal hemorrhage (Figure 4, Figure 5). Retinal nonperfusion was found in 4 patients (Figure 5), branch retinal vein occlusion in 2 patients (Figure 5), inner retinal layer hyperreflectivity in 2 patients (Figure 2), and neovascularization of the optic disk in 1 patient (Figure 4). 10 patients presented with more than 2 types of RMAs (Table 2). We finally identified 65 patients with CVD as cases and 64 patients without CVD as controls. Baseline characteristics for cases and controls are shown in Table 3. There was no difference between two groups in terms of age, sex distribution, DM, HT, HL, smoking, drinking, visual acuity and visual symptoms. BMI was significantly greater in patients with CVD (24.60±2.79) than that in patients without CVD (23.41±2.56, p=0.013). The percentage of subjects with RMAs was higher in patients with CVD compared with patients without CVD (38.5% versus 21.9%; P=0.04).

**Figure 6:**
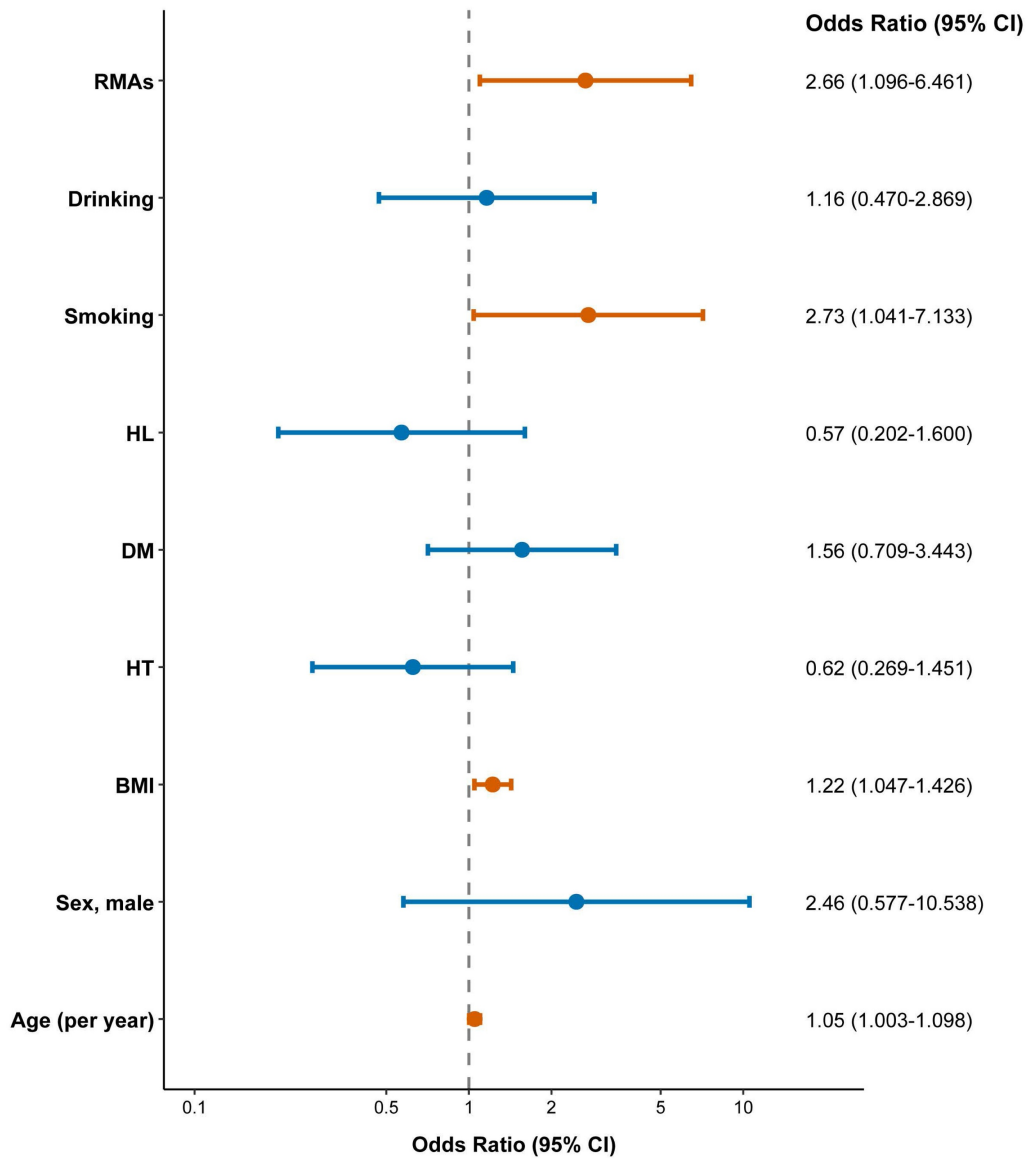
Forest plot of associations of variables to CVD.

In a multivariable logistic regression model adjusted for age, sex, BMI, hypertension, diabetes, hyperlipidemia, smoking and drinking, the presence of RMAs was significantly associated with CVD, with an OR of 2.661 (95% CI, 1.096-6.461, p=0.031; Table 4, Figure 6).

## Discussion

This study investigated and summarized the RMAs associated with severe CAS based on multimodel imaging, and found an association between the presence of RMAs and CVD in patients with severe CAS, confirming the role of RMAs as a biomarker for identifying high-risk patients with severe CAS.

It has been demonstrated in plentiful studies that stenosis of the carotid artery was associated with ocular ischemia and vessel embolization 9-11. Hayreh and colleagues investigated the presence of CAS in patients with ocular arterial occlusive disorders and found the prevalence of stenosis >50% in patients with amaurosis fugax, ocular ischemic syndrome, BRAO, CRAO and non-arteritic anterior ischemic optic neuropathy was 72%, 64%, 31%, 29%, and 11%, respectively 12. However, studies describing the ocular manifestations in patients with CAS were limited. The present study has identified a total of 39 patients who presented with RMAs evidenced by multimodel imaging. Consequently, we found a prevalence of 30.2% of RMAs in patients with severe CAS, these included RH (16.3%), Hollenhorst plaque (7.0%), BRAO (4.7%), CWS (4.7%), NP (3.1%), BRVO (1.6%), IRHF (1.6%) and NVD (0.8%). All patients with RMAs had ipsilateral lesions, and only 4 patients had bilateral lesions. It's known that systemic diseases like diabetes or hypertension affect both eyes symmetrically. So it's important to recognize that asymmetrical ocular abnormalities may be associated with CAS and should be further screened for carotid diseases, though patients may have no or only mild visual symptoms.

Ocular biomarkers have long been explored for identifying systemic diseases, and this is especially important for life-threatening diseases like CVD 13-15. Patients with CAS were at high risk of developing cardiovascular events. It's been found that increased plaque thickness, the presence or progression of carotid plaques in the common carotid artery were risk factors for CVD 16, 17. Recently, the degree of common carotid artery was proved to be a predictive marker for the development of CVD in the general population 18. However, to identify high-risk population within CAS patients is still of vital importance for medical decision-making as rapid increase of prevalence of CAS 19. The ocular vasculature shares similar anatomical and physiological characteristics with the cerebral and coronary circulations and plenty of studies have explored its significance in diagnosing and predicting CVDs 20, 21. In the present study, we utilized SS-OCTA and CFP, which has increased the accuracy and sensitivity for identifying RMAs. In the subsequent analysis, we found that RMAs was an independent risk factor for CVD in patients with severe CAS. In the management of patients with CAS, special care should be focused on the eye, and those with RMAs were at higher risk of CVD and should be carefully managed.

There are several limitations within the present study. First of all, this is a cross-sectional study, which was not able to prove the predictive value of RMAs for the development of cardiovascular events, and this should be further investigated in future studies. Secondly, some may doubt that RMAs were caused by other systemic diseases like diabetes and hypertension, other than CAS. It's difficult to quantify the impact of CAS on the eye, especially in cases when patients were complicated with diabetes or hypertension which is common in patents with CAS.

## Conclusion

This cross-sectional study summarized the RMAs associated with CAS and identified RMAs as biomarkers of CVD in patients with severe CAS. Patients with RMAs were at high risk and should be carefully managed.

## Figures and Tables

**Table 1:** Demographic characteristics of included 129 patients with severe CAS

	Patients	p-value
No.	129	
Age (years)	66.38±9.11	
Sex	115M (89.1%), 14F (10.9%)	
CVD	65 (50.4%)	
DM	53 (41.1%)	
HT	85 (65.9%)	
HL	24 (18.6%)	
Smoking	75 (58.1%)	
Drinking	62 (48.1%)	
BMI	24.0±2.73	
SBP (mmHg)	134.9±18.3	
DBP (mmHg)	81.07±10.3	
Ipsilateral BCVA	0.08±0.16	
Contralateral BCVA	0.06±0.11	p=0.113
Ipsilateral AL	23.8±0.84	
Contralateral AL	23.8±1.11	p=0.193
Ipsilateral IOP	13.5±2.8	
Contralateral IOP	14.3±3.3	p<0.001*
Visual symptom	53 (41.1%)	
AF	35 (27.1%)	
VFD	6 (4.7%)	
DVA	15 (11.6%)	

CVD: cardiovascular diseases; DM: diabetes mellitus; HT: hypertension; HL: hyperlipidemia; BMI: body mass index; SBP: systolic blood pressure; DBP: diastolic blood pressure; BCVA: best corrected visual acuity; AL: axial length; IOP: intraocular pressure; AF: amaurosis fugax; VFD: visual field defect; DVA: decreased visual acuity

**Table 2:** RMAs presented in patients with severe CAS

	Number (percentage)
Patients with RMAs	39/129 (30.2%)
Ipsilateral RMAs	39
Bilateral RMAs	4
BRAO	6/129 (4.7%)
Ipsilateral BRAO	5
Contralateral BRAO	1
ARE	9/129 (7.0%)
Ipsilateral ARE	8
Contralateral ARE	1
RH	21/129 (16.3%)
SRH	3
Ipsilateral SRH	2
Contralateral SRH	1
DRH	18
Ipsilateral DRH	17
Contralateral DRH	1
CWS	6/129 (4.7%)
Ipsilateral CWS	5
Contralateral CWS	1
Ipsilateral NP	4/129 (3.1%)
Ipsilateral IRLH	2/129 (1.6%)
Ipsilateral NVD	1/129 (0.8%)
Eyes with ≥2 RMAs	10/129 (7.8%)

RMAs: retinal microvascular abnormalities; CAS: carotid artery stenosis; BRAO: branch retinal artery occlusion; ARE: asymptomatic retinal emboli; BRVO: branch vein occlusion; RH: retinal hemorrhage; SRH: superficial retinal hemorrhage; DRH: deep retinal hemorrhage; CWS: cotton wool spots; NP: nonperfusion; IRLH: inner retinal layer hyperreflectivity; NVD: neovascularization of the optic disk

**Table 3:** Baseline characteristics of severe CAS patients with or without CVD

	Patients with CVD	Patients without CVD	p-value
No.	65	64	
Age (years)	67.42±8.24	65.33±9.87	0.194
Male	58 (89.2%)	57 (89.1)	0.975
DM	32 (49.2%)	21 (32.8%)	0.058
HT	42 (64.6%)	42 (65.6%)	0.949
HL	12 (18.5%)	12 (18.8%)	0.966
Smoking	42 (64.6%)	33 (51.6%)	0.133
Drinking	33 (50.8%)	29 (45.3%)	0.535
BMI	24.60±2.79	23.41±2.56	0.013*
Ipsilateral BCVA	0.10±0.18	0.06±0.13	0.209
Contralateral BCVA	0.08±0.11	0.05±0.10	0.124
Visual symptoms	30 (46.2%)	23 (35.9%)	0.238
AF	19 (29.7%)	16 (25.0%)	0.589
RMAs	25 (38.5%)	14 (21.9%)	0.040*

CVD: cardiovascular diseases; DM: diabetes mellitus; HT: hypertension; HL: hyperlipidemia; BMI: body mass index; BCVA: best corrected visual acuity; AF: amaurosis fugax; RMAs: retinal microvascular abnormalities; *: p<0.05

**Table 4:** Multivariable Logistic Regression Model Evaluating the Relationship Between Presence of RMAs and CVD

Variables	OR	95%CI	p-value
Age (per year)	1.050	1.003-1.098	0.035*
Sex, male	2.465	0.577-10.538	0.223
BMI	1.222	1.047-1.426	0.011*
HT	0.625	0.269-1.451	0.274
DM	1.562	0.709-3.443	0.268
HL	0.568	0.202-1.600	0.284
Smoking	2.725	1.041-7.133	0.041*
Drinking	1.161	0.470-2.869	0.747
RMAs	2.661	1.096-6.461	0.031*

RMAs: retinal microvascular abnormalities; CVD: cardiovascular diseases; OR: odds ratio; CI: confidence interval; BMI: body mass index; HT: hypertension; DM: diabetes mellitus; HL: hyperlipidemia; *: p<0.05

## Abbreviations

CVD: cardiovascular diseases
CAS: carotid artery stenosis
RMAs: retinal microvascular abnormalities
BCVA: best-corrected visual acuity
IOP: intraocular pressure
CFP: color fundus photography
OCT: optical coherence tomography
OCTA: optical coherence tomography angiography
BRAO: branch retinal artery occlusion
ARE: asymptomatic retinal emboli
BRVO: branch retinal vein occlusion
RH: retinal hemorrhage
SRH: superficial retinal hemorrhage
DRH: deep retinal hemorrhage
CWS: cotton wool spots
NP: nonperfusion
IRLH: inner retinal layer hyperreflectivity
NVD: neovascularization of the optic disk
